# Johann Gaspar Spurzheim: A Life Dedicated to Phrenology

**DOI:** 10.7759/cureus.1295

**Published:** 2017-05-30

**Authors:** Mohammad Bilal, Bryan Edwards, Marios Loukas, Rod J Oskouian, R. Shane Tubbs

**Affiliations:** 1 Seattle Science Foundation; 2 Department of Anatomical Sciences, St. George's University School of Medicine, Grenada, West Indies; 3 Swedish Neuroscience Institute; 4 Neurosurgery, Seattle Science Foundation

**Keywords:** phrenology, neuroscience

## Abstract

Loved for his empathetic nature but admired for his analytical mind, Gaspar Spurzheim (1776-1832) was a prominent contributor to phrenology, a field of neuroscience focused on studying the shapes of the skulls in relation to the activity levels of certain functional areas of the brain. This curiosity about the connection between physical characteristics and brain function was seeded in the young mind of Spurzheim when he observed that classmates with seemingly superb memories also appeared to have bright eyes. It launched Spurzheim on a journey to study the shape and function of the brain, which eventually led him to his mentor Franz Gall. Together, they classified many functional areas of the brain while touring Europe, describing their findings to audiences that filled lecture theaters to the brim. During their lectures, Gall focused on explaining findings while Spurzheim focused on dissections and demonstrations. After spending significant time with his mentor, captivating audiences and spreading the ideas of phrenology through several books and journal publications, Spurzheim left their fruitful partnership and started lecturing on his own. He toured the UK, giving lectures and dissections in London and Edinburgh, gaining universal respect for his dissection methods, writing several books and publishing articles in what were then major journals. With the aid of his wife’s artistic skills in painting and sketching many of dissections, Spurzheim’s popularity extended beyond the phrenological community. It eventually led him to Boston, where he opened this field of science in America before his death in 1832. Although phrenology has lost its popularity, Spurzheim’s contributions to our anatomical understanding of the brain survive.

## Introduction and background

The credibility of phrenology, a subject born from the extensive research carried out by Franz Josef Gall and his pupil Gaspar Spurzheim has been a cause of debate among neurological authorities. The main concept of the subject is that the brain is made up of functional parts that cause the overlying skull to protrude outwards, enabling mental capability or traits to be predicted by identifying skull shape [1]. Although the dwindling groups of people who still believe phrenology to be a valid credit to Franz Gall with the major discoveries and criticize Spurzheim for merely restating Gall’s findings, many researchers have now begun to recognize Spurzheim as a distinct contributor to phrenology and subsequent neurological discoveries.

Born in a small town near Germany, Spurzheim’s interest in theology and medicine took root when as a young child he observed that many boys in his class who surpassed him in memorizing things had bright and prominent eyes. Excited by the possibility of a link between such a physical characteristic and a mental ability, Spurzheim began to study the shape and the physiology of brain in more detail and was sent to study medicine in Vienna [2]. 

## Review

### Spurzheim's singular beliefs

It was during his time as a private tutor in Vienna that he met his future mentor Gall [2], who was conducting a course on the brain and its parts. By then, Spurzheim had already relinquished the study of the brain as a whole and was now analyzing its parts in relation to specific prominences of the cranium that signified different talents and faculties. He called it the doctrine of the mind. Franz Gall and Spurzheim became partners and Spurzheim single-handedly undertook the dissection of the brain in all ensuing lectures while Gall explained the findings.

The meticulous dissection of the brain was not Spurzheim’s only contribution. Even though his beliefs ran parallel to Gall’s, he deviated at various points and was articulate about it. He declared that there were 35 functional organs in the brain while Gall found only 27 functional organs. Unlike Gall, who thought that a certain bulge in the cranium made a person into a murderer or a thief, Spurzheim was resolute in his belief that God could never create something innately bad. He believed that people made themselves into murderers and thieves and that the mental exertions involved led to those formations on the skull. He also rejected Gall’s idea that the brain organs were determinate, declaring that man was eventually responsible for his choices [3].

### The partnership

Dissecting brains of dead animals and refraining from vivisections, Gall and Spurzheim toured Germany to demonstrate their research [3]. Gall’s inspiration came largely from the works of Johann Gottfried von Herder, a philosopher who dismissed mind-body dualism and stated that empiricism was crucial for both body and mind. Gall followed Herder’s example and decided to explore an area that was a source of much speculation, “human nature” [4]. Gall located 27 specific organs or faculties in the brain of which eight were unique to humans and 19 were in common with animals. These eight qualities were: Comparative sagacity, metaphysical spirit, wit/joking, poetic ability, goodness/moral sense/conscience, mimicry, religious instinct, and firmness/obstinacy. The faculties were also divided into two orders: ‘feelings’, which were also found in animals and ‘intellectual faculties’, which were found only in humans. The first order was located at the back of the brain and the second at the front, beginning from the hairline to under the eyes [5]. The concept was simple: certain qualities or traits led to certain formations on the surface of the skull because extensive use of those parts of brain underneath them caused enlargement. These qualities could be ascertained by cranial palpation and measurements [3]. Gall also believed that sex and the feelings related to it did not originate from the cerebrum with other such faculties but were focused in the cerebellum [6]. The talent of drawing and painting, or in Gall’s words the “faculty of color”, resulted from a harmony between the colors in one’s mind [7].

Their lectures were a success, as Gall was an exceptional lecturer and Spurzheim an exceptional demonstrator. After attending one of their performances, a German anatomist named Reil stated: “I have seen the anatomical demonstrations of the brain, made by Gall, more than I thought a man could discover in his whole life”. They gathered a strong following and Gall found in his pupil, an associate with whom he could prove more than he had ever imagined. However, because of their similar ideologies and parallel abilities, Spurzheim was soon to become both “Gall’s chief apostle and rival” [1]. Very soon, he felt he could point out a significant bump on the heads of all painters or could recognize all musicians by the shape of their heads or could tell which head foretold unique intellectual powers. With the fame they invited, came an equal share of criticism, mainly from religious and political authorities. The duo traveled to Paris and with their increasing collection of skulls, their lectures became more detailed and convincing [2]. People began openly to compare phrenology with physiognomy, the science of judging a person’s character by the face [8] and with psychoanalysis as founded by Freud [9]. Gall loathed the idea of being associated with physiognomy, declaring that physiognomy had not come upon even “one single, solid principle” [8].

### Work of Spurzheim

In 1813, Spurzheim traveled to London and gave his first lecture without Gall in the amphitheater of Abernethy. Although he could not persuade his audience towards the concept of phrenology, he set a new standard of brain dissection, earning him universal respect [3]. In a letter published in The British Journal, James George Davey reprimanded those who ridiculed phrenology and stated that “- in a word, phrenology, is a great fact…” [10]. Spurzheim’s next stop was Edinburgh, where an article had previously been published against his work in phrenology. Holding that article in one hand and dissecting a brain with the other, he won his audience over with his simple yet strong arguments [3]. He went on to win over Boston in 1832 and opened a whole new era of education in the United States [11]. His untimely death in the same year caused great sorrow among his fans and followers.

### Publications

After co-authoring the first two of the four volumes of "Anatomie et Physiologie" with Gall [12], Spurzheim published many works during his stay in England. The most notable were "The Physiognomical System" in which the chapters, he had later broken down to detailed explanation, naming the writings "Phrenology and Outlines of Phrenology." He also wrote the "Examination of the Objections" made in Great Britain against Phrenology. The investigations he made with Gall were published in "The Anatomy of the Brain." "Philosophical Principles of Phrenology" brings out Spurzheim’s religious inclination and how he relates it to his subject. The supreme importance he places on education and counseling can be deduced from his "Elementary Principles of Education." In "Insanity" he addresses derangements of the human brain [3].

In his books, Spurzheim included more faculties/organs of the brain than the 27 declared by Franz Gall. Furthermore, Spurzheim omitted the “bad” faculties assumed by Gall which led to Gall being openly critical of Spurzheim’s work as he defended his own life’s research [12].

### Marriage

Spurzheim was known to be a man of exceptional character. His kindness, humility, and empathy for others were common knowledge. It, therefore, delighted his friends and fans when he married a woman of equal qualities. A widow and a mother of three, Mrs. Spurzheim dedicated herself to the life and work of her husband. Her knowledge of science and taste in art equaled that of Spurzheim. It was her skillful sketching and painting that began to aid Spurzheim’s demonstrations and attracted people unrelated to his field. Historians today have to thank her for some of the very elaborate sketches used by Spurzheim in his classes [1-2].

The marriage nurtured Spurzheim’s mind and heart in a way that was obvious to everyone around him. The prime reason he was enthralled with her was that she had faced immense suffering which, according to him, makes a person more able to understand and alleviate other people’s griefs [5]. Perhaps this was why the addition of Mrs. Spurzheim to his career and life brought him, even more, success, fame, and most of all, inner peace. Her death left him eternally wounded. He fell ill and blamed his illness on the fact that he no longer had warm linen to look forward to when he came home as he had when his wife was alive [1]. He lived only three years after her death. 

## Conclusions

Although phrenology became much less respected as new fields of science thrived, Spurzheim contributed greatly to our early anatomical understanding of the brain through his lectures, dissections, and publications.
